# A Relational Turbulence Theory Perspective on Interpersonal Conflict During the Transition to Parenthood

**DOI:** 10.3390/ejihpe15110237

**Published:** 2025-11-18

**Authors:** Roi Estlein, Jennifer A. Theiss, Kirsten M. Weber, Hannah E. Jones

**Affiliations:** 1 School of Social Work, University of Haifa, Haifa 3498838, Israel; 2 Department of Communication, Rutgers, The State University of New Jersey, New Brunswick, NJ 08901, USA; jtheiss@rutgers.edu; 3 Department of Communication and Dramatic Arts, Central Michigan University, Mount Pleasant, MI 48859, USA; weber2km@cmich.edu; 4 Department of Communication, University of Kentucky, Lexington, KY 40506, USA; hannahjones@uky.edu

**Keywords:** conflict communication, interdependence, relational turbulence, relational uncertainty, romantic relationships, transition to parenthood

## Abstract

This study applied relational turbulence theory to examine how relationship characteristics in the form of relational uncertainty and partner interdependence during the transition to parenthood are associated with more severe appraisals of irritations, features of communication during couple conflict, and perceptions of increased turbulence in the relationship. We conducted a longitudinal study of 78 couples who were surveyed three times during the transition to parenthood from pregnancy to six months after birth. Data were analyzed using multilevel modeling and examined both actor and partner effects. Results point to between-person and within-person actor effects, with actors’ relational uncertainty and facets of interdependence predicting perceived severity of irritations and features of conflict episodes. In addition, actors’ severity of irritations predicted conflict features and perceived relational turbulence. Partner effects emerged for relational uncertainty predicting communicative openness, conflict management, and relational turbulence, and facets of interdependence predicting most outcomes. The results are discussed in terms of their theoretical contributions and practical implications for first-time parents.

## 1. Introduction

The transition to parenthood can represent a transformative stage for couples, bringing marked changes to their relationship dynamics that may challenge partners’ relational routines and communication patterns (Doss & Rhoades, 2017). Although becoming a new parent can be a very fulfilling and rewarding experience (Nelson et al., 2013), many couples are unprepared for the various stressors that emerge with the birth of a child that can take a toll on their relationship (Mihelic & Morawska, 2018). During the transition to parenthood partners need to reestablish roles and task responsibilities, reorganize the division of housework, coordinate childrearing, and renegotiate existing boundaries (Claxton & Perry-Jenkins, 2008). With the variety of changes that accompany the birth of a child, relationship partners tend to experience less emotional intimacy and empathy during this transition (Doss & Rhoades, 2017), participate in more conflict (Cowan & Cowan, 2000), and are generally less satisfied (Keizer & Schenk, 2012). Thus, the aim of this study is to employ a dyadic perspective to examine the interplay between partners’ relationship characteristics in the form of relational uncertainty and partner interdependence, interpersonal conflict, and perceptions of relational turbulence during the transition to parenthood.

We draw on the relational turbulence theory as a theoretical foundation for this study because it points to relationship characteristics that are heightened during relationship transitions and give rise to more extreme reactions to relationship events (Solomon et al., 2016). Specifically, the theory suggests that transitions are ripe for increased relational uncertainty and changes to partner interdependence, which create conditions in which individuals are more reactive to their relational circumstances (Solomon et al., 2010). In a context where couples are more sensitive to interpersonal events, partner behaviors that were previously perceived as innocuous and tolerable may now be perceived as irritating and intolerable (Theiss & Solomon, 2006). Moreover, increased reactivity between partners contributes to a relational environment that feels tumultuous and unsettled (Knobloch & Theiss, 2010). Thus, the transition to parenthood creates conditions where reactions to relationship events tend to be intensified, perceptions of irritations may be more severe, and communication about irritations becomes more intense, which may contribute to global evaluations of the relationship as turbulent.

Importantly, prior research on relational turbulence has primarily examined between-person differences, focusing on how individuals who report higher uncertainty or partner interference or facilitation tend to experience more severe irritations and turbulence overall. However, such approaches overlook potential within-person fluctuations that occur as partners navigate major relational transitions. By modeling both between- and within-person effects, the present study captures not only stable individual differences but also dynamic changes in relational uncertainty, interdependence, and conflict processes over time. This approach provides a more nuanced understanding of how relational turbulence evolves during the transition to parenthood and extends prior work that has often relied on cross-sectional or single-wave data.

The goals of this paper are twofold. First, we apply relational turbulence theory to examine how relationship conditions during the transition to parenthood are associated with more intense conflict episodes and appraisals of turmoil in the relationship. Second, we examine the extent to which actors’ and partners’ experiences of interpersonal conflict and conditions of relational turbulence are interdependent. Taken together, these goals make theoretical, methodological, and practical contributions to the literature on relational turbulence and the transition to parenthood. Theoretically, this study extends relational turbulence theory by highlighting features of conflict episodes that shape and reflect conditions of turmoil in romantic relationships during this transition and examining the ways that partners’ experiences are interdependent. Methodologically, this study takes advantage of longitudinal and dyadic data to document actor-partner effects. Practically, the results of this study have utility for aiding first-time parents in recognizing and managing conditions of relational turbulence during the transition to parenthood. In the sections that follow, we point to the mechanisms in the relational turbulence theory as predictors of heightened reactivity during the transition to parenthood; then, we report the results of a longitudinal, dyadic study of first-time parents.

### 1.1. Irritations and Conflict as Markers of Turbulence During the Transition to Parenthood

When romantic partners become parents, they experience a variety of individual and relational changes that can come with unexpected stressors (Kohn et al., 2012). New parents may notice a reduction in the amount of time they spend together as a couple, the number of leisure activities they enjoy together, and the frequency of sexual intimacy or expressions of affection (Doss & Rhoades, 2017). The birth of a new child also requires romantic partners to renegotiate their relational roles and redistribute household chores, which may invoke frustration and perceived unfairness in the division of labor (Yavorsky et al., 2015). Relationship conditions during the transition to parenthood are likely to intensify conflicts about pre-existing issues and complaints (Kluwer & Johnson, 2007), but they can also spark new sources of tension, such as disagreements about child rearing, household management, or relationship maintenance (Rodriguez & Adamsons, 2012). Thus, increased conflict during the transition to parenthood is one of the main factors contributing to a decline in relationship quality and satisfaction for first-time parents (Kluwer & Johnson, 2007). Consequently, the transition to parenthood may generate increased frustration and irritation with one’s partner, as well as more intense conflicts that can undermine relationship well-being.

The relational turbulence theory offers a useful framework for explaining how relationship mechanisms during the transition to parenthood contribute to a climate of tension and turmoil between partners. The theory argues that transitional periods give rise to relationship characteristics that promote increased reactivity to interpersonal events. Specifically, the theory nominates relational uncertainty and changes in interdependence (reflected in interference and facilitation from partners) as two mechanisms that characterize relationship transitions and may contribute to a climate of relational turbulence. *Relational uncertainty* refers to the lack of confidence people have in their perceptions of relational involvement (Knobloch & Solomon, 1999), stemming from doubts about one’s own involvement in the relationship (*self uncertainty*), questions about a partner’s relational involvement (*partner uncertainty*), and ambiguity about the status of the relationship more generally (*relationship uncertainty*). Relational uncertainty undermines the ability of individuals to process relational information, which promotes biased cognitive appraisals of interpersonal episodes, such as the perceived severity of a partner’s irritating behaviors or intensity of conflict interactions (Solomon et al., 2016). Importantly, prior research documented distinct and diverge associations between each source of relational uncertainty (i.e., self, partner, and relationship) and individual and relationship outcomes (e.g., Scott & Stafford, 2018), highlighting the need to distinctively examine the contribution of each aspect to the studied variables (see also Solomon et al., 2016). *Partner interdependence* refers to the extent to which relationship partners have influence on each other in their everyday activities, which can manifest as *interference from partners* when individuals perceive their partner as undermining personal goals and actions, or *facilitation from partners* when individuals perceive that their partner assists in the achievement of daily goals and routines (Solomon et al., 2016). Disruptions to interdependence heighten affective arousal, which intensifies emotional reactions to relational events, with interference from a partner corresponding with heightened negative emotions (e.g., Estlein et al., 2022) and facilitation from a partner corresponding with more positive affect (e.g., Goodboy & Bolkan, 2023).

Prior research has documented the consequences of relational uncertainty and disrupted interdependence for people’s cognitive, emotional, and communicative reactions to relational episodes. Both mechanisms are associated with the perceived intensity and intentionality of hurtful messages, increased negative affect and jealousy (), and decreased sexual satisfaction (e.g., Solomon & Brisini, 2019; Theiss et al., 2009). Judgments of the *severity of irritations* in a relationship is an appraisal that reflects emotional reactivity in the form of frustration, annoyance, and aggravation, as well as cognitive reactivity, in terms of the perceived threat to the relationship presented by a partner’s behavior. Prior research has demonstrated that relational uncertainty and interference from partners are positively associated with the severity of irritations in dating relationships (Priem & Solomon, 2011; Theiss & Solomon, 2006); thus, one of our aims is to replicate this finding in more committed relationships facing a rather volatile transition, and further explore how partner facilitation—and not only interference—may mitigate negative cognition and emotion in this context.

Relational turbulence theory also argues that relationship characteristics during transitions contribute to interpersonal episodes that vary in terms of communication engagement (i.e., the degree to which partners approach or avoid interaction) and communication valence (i.e., the degree to which partners employ positive or negative behaviors) (Solomon et al., 2016). In the context of conflict episodes, the openness of communication and the attempts to dominate the interaction, and the conflict management strategies employed by partners, can be reflective of the engagement and valence of communication between partners. *Open communication* is highlighted as a significant feature of conflict interaction (Domingue & Mollen, 2009). Although partners may find it difficult to interact openly about sensitive topics and may perceive such communication as threatening to the self and the relationship (Theiss & Estlein, 2014), open communication can help partners talk over their disagreements and reach resolutions (Kuriansky et al., 2010). *Communicative dominance* refers to individual attempts to control the interaction to gain influence and establish power over the other participant (Dunbar, 2004). Ongoing patterns of dominating communication reflect relational distress and are associated with decreased relationship satisfaction and a sense of disconnection (Estlein & Theiss, 2022). One study that examined conflict interactions between empty-nest couples found that relational uncertainty was associated with avoidant communication behaviors, such as indirectness, topic avoidance, and withdrawal, whereas interference from partners predicted avoidant behaviors as well as more assertive behaviors, such as increased criticism (King & Theiss, 2016). Thus, relationship characteristics in the form of relational uncertainty and partner interdependence during the transition to parenthood may correspond with conflict communication that is less open and more dominating.

*Conflict management* refers to the strategies partners employ in interaction about their disagreements, which tend to be characterized as either constructive or destructive (Gao et al., 2019). Whereas destructive conflict management includes aggressive behaviors and can lead to disruptive relationship dynamics (Cummings et al., 2008), constructive conflict management involves ways to establish cooperation by respecting the other partner’s point of view and reflecting a shared effort to solve the disagreement (Delatorre & Wagner, 2018). Partners’ efforts to enact constructive conflict behaviors are associated with decreased psychological distress and low levels of depressive symptoms (Lee et al., 2020). Relational uncertainty and interference from partners are negatively associated with a variety of relationship maintenance behaviors for romantic partners, including fewer assurances, less open communication, and more destructive conflicts (Theiss & Knobloch, 2014). Moreover, relational uncertainty and interference from partners are associated with more distributive conflict tactics, whereas facilitation from partners correlates with more integrative conflict tactics (Worley & Shelton, 2020). Therefore, the relationship characteristics that contribute to a climate of turbulence during transitions may undermine couples’ capacity for constructive conflict management.

Importantly, because romantic partners interact frequently and their relationship experiences are deeply intertwined, there is mutual influence between partners’ perceptions of the relationship, and their cognitions, emotions, and behaviors are interdependent (Estlein & Theiss, 2022; Lee et al., 2020). In this sense, individuals’ appraisals of their relationship should be associated both with their own, as well as with their partner’s perceptions, feelings, and actions (Sanford, 2006). Thus, in the current study, we employ a dyadic approach to explore the effects of both one’s own and their partner’s relational uncertainty and perceived interference and facilitation on individuals’ appraisals of conflict episodes. Indeed, Theiss and Knobloch (2014) found that both actors’ and partners’ relational uncertainty and interference from a partner contribute to a relational climate where actors’ appraisals of irritations are more severe. Thus, in light of theorizing and empirical evidence demonstrating interdependence between romantic partners’ experiences, we advance the following hypotheses to examine associations between actors’ and partners’ perceptions of relationship characteristics and features of interpersonal conflict episodes during the transition to parenthood:

**H1.**  *Actors’ and partners’ self, partner, and relationship uncertainty are positively associated with actors’ (a) perceived severity of irritations and (b) communicative dominance, and negatively associated with (c) open communication and (d) constructive conflict management behaviors during the transition to parenthood.*

**H2.**  *Actors’ and partners’ appraisals of interference from a partner are positively associated with actors’ (a) perceived severity of irritations and (b) communicative dominance, and negatively associated with (c) open communication and (d) constructive conflict management behaviors during the transition to parenthood.*

**H3.**  *Actors’ and partners’ appraisals of facilitation from a partner are negatively associated with actors’ (a) perceived severity of irritations and (b) communicative dominance, and positively associated with (c) open communication and (d) constructive conflict management behaviors during the transition to parenthood.*

### 1.2. Severity of Irritations Predicting Features of Conflict Communication

Relational turbulence theory’s propositions also presume that during times of relational transition, biased cognitive appraisals and intensified emotional reactions to interpersonal episodes shape the engagement and valence of communication behavior (Solomon et al., 2016). Research on hurt in romantic relationships indicates that the intensity of hurt and appraisals of the intentionality and relational damage of hurtful messages are associated with more direct communication with one’s partner about the episode (Theiss et al., 2009). Similarly, irritations that are severe and threaten the relationship are associated with more direct confrontations of a partner about their behavior (Theiss & Solomon, 2006). In contrast, relationship partners tend to be more indirect and engage in more topic avoidance about relationship status (Palomares & Derman, 2019) and sexual intimacy (Theiss & Estlein, 2014) when they perceive that such interactions would be threatening to the self or the relationship. In response to boundary turbulence over privacy violations, feeling hurt is positively associated with integrative communication, but also positively associated with distributive communication and distancing, whereas anger is positively associated with distributive communication and relational distancing (McLaren & Steuber, 2013). Thus, the engagement and valence of romantic partners’ conflict is associated with their emotional and cognitive reactions to the event. In light of evidence that severe hurt and irritation prompt more direct and assertive confrontations of one’s partner, we expect that the severity of irritations is positively associated with communicative dominance; however, the tendency for increased distancing and distributive communication in response to privacy violations points to a negative association between the severity of irritations and both communicative openness and conflict management behaviors. Once again, we expect that both actors’ and partners’ perceived severity of irritations are associated with actors’ communication behavior, because the episode is co-constructed and a partner’s irritations may contribute to polarized communication responses from the actor. Thus, we advance the following hypothesis:

**H4.**  *Actors’ and partners’ severity of irritations are negatively associated with actors’ (a) communicative openness and (b) constructive conflict management, and positively associated with (c) communicative dominance.*

### 1.3. Features of Conflict Episodes Predicting Relational Turbulence

Relational turbulence theory argues that repeated interpersonal episodes characterized by polarized cognitions, emotions, and communication behavior contributes to an overarching sense of the relationship as turbulent (Solomon et al., 2016). Biased cognitions and distributive communication behavior during interpersonal conflicts can have negative consequences for romantic relationship quality and satisfaction (Roloff & Soule, 2003). Research indicates that in conversations about stressors, underaccommodation from one’s partner corresponds with perceptions of increased turbulence in the relationship (Dhillon, 2023). Moreover, conflict interactions characterized by aggressive communication (Jones & Theiss, 2021) and ineffective arguing (Brisini & Solomon, 2021) are associated with increased relational turbulence. In the case of first-time parents, the broad scope and intensity of irritations create opportunities for repeated exposure to negative communication that contributes to a sense of the relationship as turbulent. Thus, we consider appraisals of irritations and communicative features of conflict episodes as predictors of relational turbulence during the transition to parenthood. Evidence suggests that perceptions of a partner’s communication behavior can be a stronger predictor of relational turbulence than one’s own communication (Brisini & Solomon, 2020); therefore, we consider the potential for actor and partner effects in our analyses. This logic is formalized in the following hypothesis:

**H5.**  *Actors’ and partners’ (a) communicative dominance are positively associated with actors’ relational turbulence, whereas (b) communicative openness and (c) constructive conflict management are negatively associated with relational turbulence.*

## 2. Materials and Methods

To investigate our hypotheses, we recruited dyads to participate in a longitudinal study in which they completed online questionnaires at three time points during the transition to parenthood: (a) during pregnancy, (b) three months after birth, and (c) six months after birth. The study was approved by the Rutgers University’s Institutional Review Board. Recruitment was completed by posting announcements at local gynecology and obstetrics offices and in online forums for pregnant women. Individuals who were interested in participating were instructed to email the researchers, at which point they received a brief screening questionnaire to verify that they met the eligibility requirements for the study. Individuals were eligible to participate if (a) they were involved in a heterosexual romantic relationship, (b) they or their partner were pregnant with their first child, (c) the pregnancy had progressed to the second or third trimester, (c) both individuals in the relationship were the biological parents of the child, (d) the partners were cohabiting, and (e) both partners were at least 18 years of age. When eligibility was confirmed, we requested contact information for the romantic partner to obtain informed consent, and each individual was assigned a unique username and password to access the online questionnaires. Individuals received a $15 gift card to a national retailer for completing the first and last waves of the study and a $10 gift card for completing the second wave.

### 2.1. Participants

The sample consisted of 78 heterosexual couples (156 individuals). The response rate across all waves of the study ranged from 97% to 77%. The average age of participants was 28.38 years (range 18 to 48 years). Most of the participants in the study were Caucasian (83.2%), and the remaining participants were Hispanic (5.8%), Indian (3.9%), African American (3.2%), Native American (3.2%), Asian/Pacific Islander (1.9%), and the rest of the participants identified as Other (2.6%) The relationship status of the couples included 68 married, six engaged, and four cohabiting but not married. Couples had been in their current relationship status for an average of 2.47 years (range = 3 moths to 10 years). The majority of pregnancies were planned (67.09%).

### 2.2. Procedures

Participants completed an online survey for each wave of the study. The pre-birth questionnaire obtained demographic and relational information. In all three waves of surveys, participants completed Likert-type scales to rate their relational uncertainty, perceptions of partner interference and facilitation, appraisals of irritations, communicative features of a recent conflict, and relational turbulence. They also completed an open-ended question listing the various irritations they had with their partner in the previous week and rating the severity of each irritation.

### 2.3. Measures

Confirmatory factor analyses (CFA) were conducted on all multi-item scales to ensure they met the criteria of face validity, internal consistency, and parallelism (Hunter & Gerbing, 1982). Criteria for acceptable model fit were χ^2^/df < 3, confirmatory fit index (CFI) > 0.95, and root mean squared error of approximation (RMSEA) < 0.08 (Kline, 2023). After confirming unidimensionality, composite scores were calculated for each scale by averaging responses across items.

**Relational uncertainty.** We used Knobloch’s (2008) scale that measures the sources of relational uncertainty in marriage. Respondents used a six-point Likert-type scale (1 = *completely or almost completely uncertain*, 6 = *completely or almost completely certain*) to respond to items that were preceded by the stem, “How certain are you about…?” All items were reverse coded so that the resulting measure indexed relational uncertainty. Each member of the dyad independently completed all items of the scale, reporting on their own perceptions of self, partner, and relationship uncertainty. These assessments were completed separately by both partners and analyzed as distinct actor and partner scores in the dyadic models.

The *self uncertainty* scale consisted of four items (e.g., “your feelings about your relationship”) (*M =* 1.26; *SD* = 0.51; α = 0.92). *Partner uncertainty* was measured with four items (e.g., “your partner’s view of your relationship”) (*M =* 1.65; *SD* = 0.80; α = 0.92). The *relationship uncertainty* scale included four items (“e.g., “the current status of your relationship”) (*M =* 1.30; *SD* = 0.50; α = 0.77).

**Interference and facilitation from partners.**Solomon and Knobloch’s (2001) scale was used to measure partner interference and facilitation. Participants used a six-point Likert-type scale (1 = strongly disagree, 6 = strongly agree) to indicate their level of agreement with five items for both partner interference and facilitation. An example item for partner interference includes “this person interferes with the achievement of everyday goals I set for myself” (*M =* 1.60; *SD* = 0.87; α = 0.85). An example item for partner facilitation includes “this person helps me in my efforts to make plans” (*M =* 4.95; *SD* = 0.99; α = 0.90).

**Sources of irritations.** Participants responded to open-ended questions designed to assess perceived irritations or irritating behaviors enacted by their partner during the transition to parenthood. Participants were instructed, “In the space provided below, please describe one behavior or personality characteristic of your partner that causes you to feel irritated or annoyed, or that causes conflict in your relationship.” Participants reported 0 to 3 irritations, with an average of 2.72 irritations per spouse in Wave 1, 1.37 irritations per spouse in Wave 2, and 1.39 irritations in Wave 3.

**Perceived severity of irritations.** Following Theiss and Solomon (2006), after identifying an irritation, participants were then asked to use Likert scales (1 = strongly disagree, 7 = strongly agree) to evaluate the degree to which the behavior or characteristic was a problem and to what degree the behavior or characteristic threatens the relationship. A composite measure of the severity of irritations was calculated by averaging the ratings for all irritations that were identified by the participant (*M* = 3.37, *SD* = 1.43, α = 0.90).

**Communicative openness and dominance.** Communication openness and dominance were measured with Theiss and Knobloch’s (2014) scale. Participants used a seven-point Likert-type scale (1 = strongly disagree, 7 = strongly agree) to indicate their level of agreement with five items each for openness and dominance. An example item for communicative openness includes “I have freely disclosed my opinions to my partner” (*M =* 5.87; *SD* = 1.11; α = 0.82). An example item for communicative dominance includes “I have been argumentative with my partner” (*M =* 3.07; *SD* = 1.49; α = 0.85).

**Conflict management.** The conflict management subscale from Stafford et al.’s (2000) relationship maintenance scale was used to measure how relational partners manage conflict. Participants used a seven-point Likert-type scale (1 = not at all, 7 = very often) to indicate how often they utilized specific behaviors in their relationship (e.g., “apologize to your partner when you’re wrong”) (*M =* 5.94; *SD* = 0.84; α = 0.75).

**Perceptions of turbulence.** A scale developed by Knobloch (2007) solicited people’s appraisals of turbulence. Individuals rated a series of adjectives completing the stem, “At the present time, this relationship is …” (1 = strongly disagree, 6 = strongly agree). Seven items formed a unidimensional factor: (a) chaotic, (b) turbulent, (c) in turmoil, (d) tumultuous, (e) frenzied, (f) overwhelming, and (g) stressful (*M* = 1.71, *SD* = 0.98, α = 0.91).

### 2.4. Data Analysis

Hierarchical Linear Modeling (HLM) software version 6.8 was used to evaluate hypotheses because it is designed to accommodate nested data. We constructed three-level models using maximum likelihood estimation with repeated measures (e.g., relational uncertainty, partner interference, severity of irritations, communication features) as Level 1 variables, stable individual variables (e.g., age, sex) as Level 2 variables, and dyadic characteristics (e.g., length of relationship) as Level 3 variables. Level 1 predictors were entered into the model as group-mean-centered variables (i.e., centered around the individual’s mean across waves) to examine within-person effects. In addition, we computed individuals’ means for each variable across waves, which were entered as Level 2 variables on the intercept for each model to examine between-person effects. All models included both actors’ and partners’ scores as within-person and between-person predictors to assess actor-partner interdependence. All slopes were estimated as fixed effects and intercepts were estimated as random effects.

## 3. Results

### 3.1. Relationship Characteristics Predicting Features of Conflict Episodes

The first set of analyses examined relational uncertainty, partner interference, and partner facilitation as predictors of the perceived severity of communicative features of conflict interaction (see Table 1 for a summary of all associations). Beginning with severity of irritations as the outcome, the between-person results on the intercept indicated that, relative to others in the sample, actors’ self, partner, and relationship uncertainty and interference from partners were positively associated with the severity of irritations, whereas actors’ perceived facilitation from partners was negatively associated with irritation severity. In addition, a significant partner effect emerged, such that actors reported more severe irritations when their partner perceived greater interference relative to other partners in the sample. Turning to the within-person effects, which are indicated in the slopes of the model, all three sources of actors’ relational uncertainty and interference from partners were positively associated with the severity of irritations, and facilitation from partners was negatively associated with the severity of irritations. These results are relative to the individual’s average level of each variable across waves, such that severity of irritations was heightened during waves when actors were experiencing higher than their average levels of relational uncertainty or partner interference and lessened when receiving more partner facilitation than is typical. There were also within-person partner effects for interference and facilitation from partners, such that actors reported more severe irritations during waves when their partner was experiencing higher levels of partner interference than normal, and less severe irritations during waves when their partner reported higher than average partner facilitation.

We then looked at actors’ and partners’ relationship characteristics as predictors of communicative openness, communicative dominance, and conflict management behavior. The between-person effects on the intercept showed that actors’ relational uncertainty and interference from partners were negatively associated with communicative openness and conflict management behavior and positively associated with communicative dominance relative to others in the sample. Actors’ facilitation from partners was positively associated with openness and conflict management between persons, but there was not a significant between-persons effect of partner facilitation on communicative dominance. Two between-persons partner effects emerged, such that actors reported more communicative dominance when their partner perceived more interference relative to other partners in the sample, and actors reported more constructive conflict management when their partner experienced relatively more facilitation.

Within-person results showed that during waves when actors and partners reported more relational uncertainty and partner interference than was typical for their relationship, actors reported less openness in conflict communication. Actors’ facilitation from partners was positively associated with communicative openness, but the effect for partners was nonsignificant. Communicative dominance was only predicted as within-person actor effects, such that actors’ relational uncertainty and interference from partners were positively associated with dominance in conflict communication, and actors’ facilitation from partners was negatively associated with communicative dominance. Finally, constructive conflict management behavior was negatively associated with actors’ self, partner, and relationship uncertainty, and with the partners’ reports of self and partner uncertainty. Thus, actors reported less constructive conflict management tactics during waves when they and their partner both reported higher than average levels of relational uncertainty. In addition, actors’ reports of facilitation from partners were positively associated with conflict management behavior, such that waves with higher-than-normal levels of facilitation corresponded with more effective conflict management.

### 3.2. Severity of Irritations Predicting Communicative Features of Conflict Episodes

Our next set of analyses examined actors’ and partners’ perceived severity of irritations as predictors of actors’ reported communicative openness, communication dominance, and conflict management behaviors during conflict episodes (see Table 2). Between-person effects on the intercept showed that actors with more severe irritations relative to others in the sample reported less communicative openness and constructive conflict behaviors and more communicative dominance during conflict interactions. There were no significant between-person partner effects. A similar pattern emerged for the within-person effects, such that during waves when actors reported more severe irritations than usual, they showed less communicative openness, more communicative dominance, and less positive conflict management behaviors. In addition, partners’ severity of irritations was negatively associated with actors’ communicative openness, such that actors were less open in their conflict communication during waves when their partner was experiencing more severe irritations than was typical.

### 3.3. Features of Conflict Episodes Predicting Relational Turbulence

Our final set of analyses predicted actors’ appraisals of relational turbulence from their own and their partner’s severity of irritations and conflict communication behaviors (see Table 3). Between-person effects on the intercept demonstrated that actors reported heightened relational turbulence when they experienced more severe irritations and dominant communication, and less communicative openness and conflict management, relative to others in the sample. In addition, actors’ relational turbulence was heightened when their partner reported engaging in more communicative dominance compared to other partners. Within-person effects indicated that actors perceived more relational turbulence during waves when they experienced higher than average levels of irritation severity and communicative dominance, and lower than average levels of open communication and constructive conflict management. The within-person partner effects were nonsignificant.

## 4. Discussion

The transition to parenthood in romantic relationships brings about a variety of changes to partners’ individual and relational roles and routines. As partners attempt to make sense of their circumstances and coordinate new behavioral patterns in their relationship after the birth of a child, they may experience a variety of frustrations and irritations that can erupt into more volatile conflict interactions and a more tumultuous relational climate. This study drew on the mechanisms in the relational turbulence theory (Solomon et al., 2016) to predict the severity of irritations, communicative features of couple conflict, and perceptions of relational turbulence during the transition to parenthood. Theoretically, this study extends relational turbulence theory by adding to a growing body of literature that explores how romantic partners’ experiences of interpersonal conflict and relational turmoil are interrelated, revealing the potential for interdependence as each partner’s cognition and communication behavior are associated with outcomes for the other. Methodologically, the longitudinal and dyadic design of this study enabled an examination of associations as between-person effects, which is typical of tests of relational turbulence theory, but also as within-person effects that vary across the span of this relational transition, highlighting the dynamic nature of relational turbulence processes over time. Practically, the results of this study illuminate the sources and outcomes of irritations and intense conflicts that romantic partners may experience during the transition to parenthood, which offers several points of intervention to assist couples in navigating this event with less relational turbulence. In the sections that follow, we reflect on the contributions of this study for expanding relational turbulence theory, we highlight the implications of our findings for helping first-time parents navigate the highs and lows of the transition to parenthood, and we discuss the strengths, limitations, and future directions of this research.

### 4.1. Implications for Relational Turbulence Theory

We looked to relational turbulence theory for guidance in terms of identifying relationship characteristics that are salient during transitions and associated with increased reactivity to interpersonal events, namely, relational uncertainty and disrupted interdependence (Solomon et al., 2016). Consistent with relational turbulence theory and its recent extensions (e.g., Brisini & Solomon, 2021; Dhillon, 2023; Goodboy & Bolkan, 2023), our results showed that actors’ relational uncertainty and interference or facilitation from a partner were routinely associated with the severity of irritations and communicative features of conflict episodes. Consistent with theorizing, our findings show that heightened relational uncertainty and uncoordinated behavioral routines during relationship transitions correspond with more volatile interpersonal episodes, specifically in the form of more severe cognitive appraisals of irritations and more distributive conflict tactics. Prior research indicates that romantic partners tend to experience more frequent and intense forms of conflict during the transition to parenthood (e.g., Huss & Pollmann-Schult, 2020), which can be a driver for decreased relationship satisfaction during this time (Kluwer & Johnson, 2007), but rarely identify the circumstances that may contribute to increased conflict between partners. Our findings point to relational uncertainty and interference from partners as relationship characteristics that are heightened during transitions and may create conditions ripe for more severe interpersonal conflicts. Conversely, partners who enjoy increased facilitation during this transition are likely to benefit from more constructive conflicts. These results extend work on relational turbulence theory by looking beyond the severity of irritations as an outcome of these relationship characteristics (e.g., Solomon & Knobloch, 2004; Theiss & Solomon, 2006) to consider how the communication dynamics of conflict episodes may also be shaped by relationship conditions conducive to turbulence.

Partner effects for the relationship mechanisms predicting conflict outcomes were less common, but they emerged most strongly in terms of predicting actors’ communicative openness during conflict, as well as some significant effects for partners’ perceptions of interference and facilitation predicting actors’ conflict outcomes. First, actors reported engaging in less open communication about their irritations during waves when their partner reported higher than average levels of partner or relationship uncertainty. Similarly, we saw that during waves when a partner reported higher than typical levels of self and partner uncertainty, the actor engaged in less constructive conflict management tactics. We suspect that when individuals are experiencing doubts about the relationship, they likely behave in ways that betray these underlying questions (Li & Solomon, 2023). Individuals may be more reluctant to approach their partner to openly discuss irritations or problems if they perceive that the partner is questioning their relational involvement and may be turned off by potential confrontation or criticism. We also saw that actors reported more severe irritations, less openness, and more dominance in their conflict communication when their partner was experiencing increased interference. When partners were experiencing heightened facilitation, however, actors reported less severe irritations and more constructive conflict management tactics. Thus, individuals may attempt to tread lightly regarding conflicts if they sense that their partner is frustrated by disrupted patterns of behavior, so as not to exacerbate their partner’s annoyance. Along these lines, the partner’s severity of irritations was also negatively associated with the actor’s communicative openness, suggesting that there may be a chilling effect on people’s expression of complaints when they perceive that their partner is particularly irritated or annoyed (Li & Samp, 2019). Conversely, when partners feel that their behaviors are well coordinated, actors may be more comfortable broaching sensitive topics because their partner seems more receptive to their influence. Taken together, these findings illustrate how romantic partners’ perceptions and actions are intertwined, suggesting that individuals’ communication behavior is shaped both by their own relational appraisals as well as those of their partner.

Regarding the features of interpersonal conflict episodes that are associated with a more turbulent relationship climate, results pointed primarily to actor effects. Between person effects showed that individuals who reported more severe irritations, less open communication, and more dominant communication relative to others in the sample also experienced higher levels of relational turbulence. Within person effects indicated that individuals reported increased relational turbulence during waves when their irritations were more severe, conflict communication was less open and more dominant, and conflict management tactics were less constructive than was typical for them. Only one partner effect emerged, indicating that actors reported higher levels of relational turbulence when their partner engaged in more communication dominance relative to other partners in the sample. These results draw increased focus on the significance of interpersonal episodes for shaping the turbulent relationship environment (Solomon et al., 2016). We highlight cognitive features of conflict episodes, as well as the engagement and valence of communication about conflict, as qualities of interaction that may contribute to a sense of turmoil in the relationship. Although our analyses capture the associations between conflict episodes and relational turbulence within waves, theorizing would suggest that repeated interactions marked by these cognitive and communicative qualities coalesce over time into perceptions of relational turbulence (Solomon et al., 2019). Consistent with this view, couples who enact consistently destructive conflict behaviors over the first two years after childbirth tend to report increased depressive symptoms and more negative views of their relationship (Houts et al., 2008). Thus, the tenor of conflict interactions between partners during the transition to parenthood may be a significant factor in shaping the general climate in the relationship.

Beyond confirming patterns that have been documented in prior research on relational turbulence, the current study makes several theoretical advancements. First, by applying relational turbulence theory to the transition to parenthood—a developmental stage that has received limited empirical attention from a relational turbulence perspective—we extend the theory to a major life event that inherently alters partners’ relational routines and interdependence structures. Second, our use of a longitudinal dyadic design introduces a dynamic and interactive perspective on relational turbulence. Examining both actor and partner effects over time demonstrates that the processes of relational uncertainty, partner interference and facilitation, and conflict communication are not confined to individuals but unfold interdependently between partners. This approach refines relational turbulence theory by emphasizing its reciprocal and evolving nature within couples as they navigate a period of substantial change.

### 4.2. Practical Implications for Romantic Partners Navigating the Transition to Parenthood

Research tends to consistently show a decline in marital satisfaction over the transition to parenthood (Kluwer, 2010), which may be a reflection of increased conflict and relational turbulence arising in the months following childbirth (Huss & Pollmann-Schult, 2020). One way to forestall declining relational satisfaction is to forewarn couples about the various ways their relationship might change throughout this transition. In particular, anticipating increased uncertainty about their relational roles and disruptions to their interpersonal routines, and recognizing that these are normal experiences during the transition to parenthood, may dampen individuals’ reactivity to irritations and other relationship tensions (Knobloch et al., 2021). Similarly, reminding partners that these circumstances in their relationship, coupled with stress and exhaustion from childcare, may trigger more intense and volatile emotions can prepare them for the emotional roller-coaster that often accompanies the transition to parenthood. Thus, anticipating and normalizing the upheaval that occurs in romantic relationships after the birth of a child can be important for helping couples to interpret turmoil in their relationship in a more understanding and optimistic light.

Furthermore, the ability to communicate effectively about relational problems is associated with increased relationship satisfaction prior to becoming parents and it mitigates the decline in satisfaction after the baby’s arrival (Cox et al., 1999). Thus, one of the things that couples can do to prepare for the arrival of a baby is to arm themselves with effective communication skills, particularly around interpersonal conflict (see also Estlein & Shai, 2023). One intervention-based study demonstrated improvement in couples’ conflict communication following a psycho-educational workshop during pregnancy, with less contempt and more positive affect in husbands’ conflict communication beginning at three months post-birth and continued improvement in conflict behavior through the first year of parenthood (Shapiro et al., 2015). In this study, increased positive affect in husbands’ conflict behavior three months after birth also corresponded with more positive affect and less negative affect in wives’ conflict communication at one year postpartum. Thus, a fruitful avenue for intervention may be to encourage participation in pre-birth couples’ workshops or counseling that develop effective and constructive skills for managing conflict communication after the arrival of the baby.

### 4.3. Limitations and Future Direction

There are some limitations to this study. First, although we successfully captured part of the transition to parenthood up to the sixth month after birth, our study falls short in terms of documenting the full length of this transition and the various relationship changes partners experience as they navigate different turning points in the parenthood trajectory. Future studies should adopt a more long-term view to assess the changes that occur in romantic relationships beyond six months after birth. Second, our sample was fairly homogeneous and limited in terms of its generalizability, because it was predominantly white and only included heterosexual couples who were the biological parents of the child. Certainly, couples with different backgrounds and unique circumstances around their pregnancy may experience the transition to parenthood differently. Future research should strive for greater diversity and explore the experiences of first-time parents in other family settings, such as adoptive and same-sex couples. Relatedly, information on participants’ education and household income was not collected, which limited our ability to characterize the socioeconomic composition of the sample. Future studies should include these variables to better capture the socioeconomic context that may be associated with couples’ adaptation to parenthood. Third, although this study focused on heterosexual couples, we did not examine potential gender differences in the actor–partner effects identified in our analyses. Our goal was to document the overall dyadic patterns linking relational uncertainty, interdependence, and conflict processes during the transition to parenthood, rather than to differentiate between mothers’ and fathers’ experiences. Given the modest sample size, conducting separate analyses by gender would have substantially reduced statistical power and the stability of parameter estimates. Moreover, unlike cross-sectional SEM models that can estimate effects for husbands and wives simultaneously, the longitudinal HLM approach used in this study would require separate models for each gender, further limiting power. Nevertheless, future research should explore whether and how these actor-partner associations vary between mothers and fathers by employing larger and more diverse samples. Finally, although the longitudinal design enabled us to examine within-person fluctuations across three points in the transition to parenthood, the analyses do not allow precise inferences about the timing or shape of these changes. The multilevel models capture variability around individuals’ average levels across time rather than modeling systematic trajectories at specific stages of the transition. Nonetheless, this approach provides valuable evidence of dynamic processes within couples as they adapt to parenthood. Future research could build on this work by using analytic strategies that capture more detailed temporal trajectories, such as latent growth curve modeling or intensive longitudinal designs, to further illuminate how relational turbulence evolves over time.

## 5. Conclusions

Romantic relationships undergo a variety of changes when partners become parents. Navigating this transition can be joyful, but also tumultuous. This study drew on relational turbulence theory (Solomon et al., 2016) to identify relationship characteristics that are heightened during the transition to parenthood, and it examined how these qualities may contribute to more volatile interpersonal conflict episodes and an overarching sense of relational turbulence. The results of this study pointed to interpersonal conflicts as sites where the upheaval that is felt in the relationship during this transition becomes manifest and contributes to perceptions of tumult in the relationship. Preparing couples to anticipate changes to their relationship and arming them with effective conflict management strategies may help them to successfully navigate this transition and seamlessly incorporate their new identities as parents into their broader relational roles and routines.

## Figures and Tables

**Table 1:** Actor-Partner Effects of Relationship Characteristics on Conflict Outcomes.

	Severity of Irritations		Communicative Openness		Communicative Dominance		Conflict Management Tactics	
	Actor Effect	Partner Effect	Actor Effect	Partner Effect	Actor Effect	Partner Effect	Actor Effect	Partner Effect
Intercept (between-person effects)	3.32 ***	3.31 ***	5.87 ***	5.87 ***	3.06 ***	3.06 ***	5.92 ***	5.93 ***
Self Unc.	1.52 ***	0.16	−0.86 ***	0.02	0.79 **	0.13	−0.73 ***	−0.21
Partner Unc.	0.87 ***	0.22	−0.48 ***	−0.14	0.57 ***	−0.11	−0.52 ***	−0.06
Rel. Unc.	1.31 ***	0.10	−0.83 ***	−0.02	0.64 **	0.10	−0.67 ***	−0.21
Interference	0.85 ***	0.28 **	−0.32 **	−0.01	0.41 **	0.28 *	−0.30 ***	−0.09
Facilitation	−0.75 ***	−0.14	0.39 ***	0.14	−0.18	−0.17	0.33 ***	0.20 **
Slopes (within-person effects)								
Self Unc.	0.72 ***	0.20	−0.70 ***	−0.72 ***	0.55 ***	−0.03	−0.39 ***	−0.17 *
Partner Unc.	0.40 **	−0.07	−0.41 ***	−0.21 *	0.26 *	0.17	−0.27 ***	−0.15 *
Rel. Unc.	0.95 ***	0.07	−0.53 ***	−0.48 **	0.61 **	−0.20	−0.45 ***	−0.04
Interference	0.59 ***	0.26 *	−0.37 ***	−0.37 ***	0.36 ***	0.08	−0.08	−0.07
Facilitation	−0.43 ***	−0.21 *	0.44 ***	0.14	−0.23 ***	−0.16	0.22 ***	0.03

*Note*. Cell entries are unstandardized coefficients. Values entered under the intercept reflect the change in the intercept due to the actor’s or partner’s mean across waves of the relationship characteristic and are between-person effects. Values entered under slopes are within-person effects and reflect the change in severity of irritations during waves when the relationship characteristic was higher or lower than the person’s average across waves. Self Unc. = Self Uncertainty; Partner Unc. = Partner Uncertainty; Rel. Unc. = Relationship Uncertainty. * *p* < 0.05, ** *p* < 0.01, *** *p* < 0.001.

**Table 2:** Actor-Partner Effects of Severity of Irritations Predicting Conflict Communication.

	Communicative Openness	Communicative Dominance	Conflict Management
Intercept (between-person effects)	5.86 ***	3.16 ***	5.94 ***
A’s Irritation Severity	−0.17 **	0.47 ***	−0.27 ***
P’s Irritation Severity	−0.07	0.11	−0.00
Slopes (within-person effects)			
A’s Irritation Severity	−0.33 ***	0.14 *	−0.10 *
P’s Irritation Severity	−0.16 *	0.00	0.03

*Note*. Cell entries are unstandardized coefficients. Values entered under the intercept reflect the change in the intercept due to the actor’s or partner’s mean irritation severity across waves and are between-person effects. Values entered under slopes are within-person effects and reflect the change in the communication outcome during waves when irritation severity was higher or lower than the person’s average across waves. * *p* < 0.05, ** *p* < 0.01, *** *p* < 0.001.

**Table 3:** Actor-Partner Effects of Features of Conflict Episodes Predicting Relational Turbulence.

	Severity of Irritations		Communicative Openness		Communicative Dominance		Conflict Management Tactics	
	Actor Effect	Partner Effect	Actor Effect	Partner Effect	Actor Effect	Partner Effect	Actor Effect	Partner Effect
Intercept (between-person effects)	1.65 ***	1.65 ***	1.65 ***	1.65 ***	1.65 ***	1.65 ***	1.65 ***	1.65 ***
Severity of Irritations	0.38 ***	0.01						
Communicative Openness			−0.16 *	−0.04				
Communicative Dominance					0.17 ***	0.12 **		
Conflict Management							−0.15	−0.10
Slopes (within-person effects)								
Severity of Irritations	0.23 ***	0.08						
Communicative Openness			−0.23 ***	−0.05				
Communicative Dominance					0.15 ***	−0.01		
Conflict Management							−0.24 ***	−0.12

*Note*. Cell entries are unstandardized coefficients. Values entered under the intercept reflect the change in the intercept due to the actor’s or partner’s mean irritation severity across waves and are between-person effects. Values entered under slopes are within-person effects and reflect the change in relational turbulence during waves when features of conflict episodes were higher or lower than the person’s average across waves. * *p* < 0.05, ** *p* < 0.01, *** *p* < 0.001.

## Data Availability

The datasets generated and analyzed during the current study are available from the first or second author on reasonable request.
